# Novel insight into the detailed myocardial motion and deformation of the rodent heart using high-resolution phase contrast cardiovascular magnetic resonance

**DOI:** 10.1186/1532-429X-15-82

**Published:** 2013-09-14

**Authors:** Emil KS Espe, Jan Magnus Aronsen, Kristine Skårdal, Jürgen E Schneider, Lili Zhang, Ivar Sjaastad

**Affiliations:** 1 Institute for Experimental Medical Research, Oslo University Hospital and University of Oslo, Kirkeveien 166, N-0407, Oslo, Norway; 2 KG Jebsen Cardiac Research Center and Center for Heart Failure Research, University of Oslo, Oslo, Norway; 3 Bjørknes College, Oslo, Norway; 4 Department of Cardiovascular Medicine, University of Oxford, Oxford, UK

**Keywords:** CMR, 3D phase contrast, Strain analysis, Tissue phase mapping, Myocardial motion, Myocardial strain, Motion artifacts

## Abstract

**Background:**

Phase contrast velocimetry cardiovascular magnetic resonance (PC-CMR) is a powerful and versatile tool allowing assessment of *in vivo* motion of the myocardium. However, PC-CMR is sensitive to motion related artifacts causing errors that are geometrically systematic, rendering regional analysis of myocardial function challenging. The objective of this study was to establish an optimized PC-CMR method able to provide novel insight in the complex regional motion and strain of the rodent myocardium, and provide a proof-of-concept in normal and diseased rat hearts with higher temporal and spatial resolution than previously reported.

**Methods:**

A PC-CMR protocol optimized for assessing the motion and deformation of the myocardium in rats with high spatiotemporal resolution was established, and ten animals with different degree of cardiac dysfunction underwent examination and served as proof-of-concept. Global and regional myocardial velocities and circumferential strain were calculated, and the results were compared to five control animals. Furthermore, the global strain measurements were validated against speckle-tracking echocardiography, and inter- and intrastudy variability of the protocol were evaluated.

**Results:**

The presented method allows assessment of regional myocardial function in rats with high level of detail; temporal resolution was 3.2 ms, and analysis was done using 32 circumferential segments. In the dysfunctional hearts, global and regional function were distinctly altered, including reduced global peak values, increased regional heterogeneity and increased index of dyssynchrony. Strain derived from the PC-CMR data was in excellent agreement with echocardiography (r = 0.95, p < 0.001; limits-of-agreement −0.02 ± 3.92%strain), and intra- and interstudy variability were low for both velocity and strain (limits-of-agreement, radial motion: 0.01 ± 0.32 cm/s and −0.06 ± 0.75 cm/s; circumferential strain: -0.16 ± 0.89%strain and −0.71 ± 1.67%strain, for intra- and interstudy, respectively).

**Conclusion:**

We demonstrate, for the first time, that PC-CMR enables high-resolution evaluation of *in vivo* circumferential strain in addition to myocardial motion of the rat heart. In combination with the superior geometric robustness of CMR, this ultimately provides a tool for longitudinal studies of regional function in rodents with high level of detail.

## Background

The intricate motion and deformation of the heart can be assessed *in vivo* with varying degree of detail using several different techniques, including sonomicrometry, echocardiography employing Tissue Doppler Imaging or speckle-tracking strain analysis, as well as various cardiovascular magnetic resonance (CMR) techniques. Compared to other modalities, CMR offers measurements of true 3D function with practically no limitations in visualization geometry, and thus provides complete freedom in choosing regions for examination. Different techniques for the evaluation of myocardial function are available, including myocardial tagging [1], strain-encoded CMR (SENC) [2], displacement-encoded imaging with stimulated echoes (DENSE) [3] and phase contrast imaging (PC-CMR) [4]. The latter two offer pixel-wise measurement of displacement and velocity, respectively, allowing for high-resolution evaluation of tissue function. However, PC-CMR is the only CMR technique that been shown to allow assessment of velocity with high spatial and temporal resolution [5] and, subsequently, displacement [6], strain rate [7] and strain [8] concurrently, throughout the entire cardiac cycle. While PC-CMR velocimetry is emerging as a powerful and versatile tool for assessment of tissue motion both in humans and in rodents [5,9], it has only been reported so far for the assessment of average myocardial velocities within a slice, or in a few (<=8) circumferential segments. Also, it might be challenging to achieve the optimal temporal resolution to capture the fine details of cardiac motion in small animals. Thus, to accurately investigate the regional function e.g. in hearts with infarctions with various sizes, and in order to derive parameters such as subtle dyssynchrony, transmural functional gradients or longitudinal spread of dysfunction, data with higher spatial and temporal resolution along with appropriate post-processing procedures are essential.

**Figure 1:**
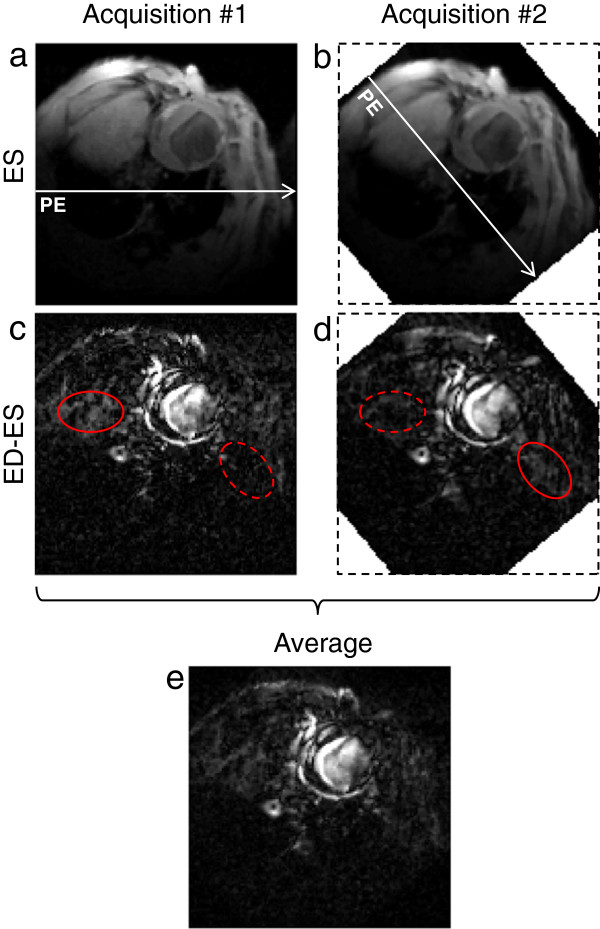
**Geometrically systematic artifacts.** Even after black-blood preparation, some flow artifacts remain. Although not obvious in the original end-systolic (ES) magnitude images **(a**,**b)**, the *difference* between the magnitude images of end-diastole (ED) and end-systole **(c**,**d)** reveal geometrically systematic artifacts. A rotation of the field-of-view alters the characteristics of these artifacts. Both acquisitions exhibit unique artifacts in the phase-encoding direction (solid ellipses), not present in the other (dashed ellipse). In the average image **(e)**, these artifacts are reduced. For illustrative purposes, a median filter has been applied for noise reduction in this figure. In **c**-**e** the grayscale has been exaggerated for clarity.

In PC-CMR, the displacement of spins between the centers of the bipolar encoding gradient lobes is encoded into the phase of the MR signal, producing datasets with near-instantaneous velocities with temporal resolution equal to the TR [10,11]. Although PC-CMR is prone to errors from several sources, including concomitant gradient [12] and eddy-current induced [13] artifacts, several approaches has been proposed to minimize these errors [12,14,15]. However, PC-CMR encoded acquisitions are intrinsically non-motion compensated and thus particularly sensitive to flow- and motion related artifacts, such as ghosting due to beat-to-beat variation in blood flow [16,17]. In Cartesian imaging, motion-related artifacts manifest in the phase-encoding direction, and may (in a short-axis view) affect the measurements non-uniformly over the circumference of the myocardium. This could lead to systematic errors when regional myocardial function is to be assessed, reducing the effective level of detail available for analysis. Black-blood contrast is therefore essential in PC-CMR of the myocardium, reducing this effect [5]. In addition, the impact of directionally dependent artifacts in studies employing signal averaging can be reduced by an in-plane rotation of the field-of-view (FOV) between the acquisitions, referred to as rotating FOV (Figure 1).

In this study, we present a PC-CMR approach for assessing left ventricular (LV) myocardial motion in rats, employing a rotating FOV along with optimized acquisition parameters and post-processing protocols. We aim to demonstrate the feasibility of PC-CMR for accurately describing both myocardial motion and deformation in rodents by deriving global parameters such as peak velocities and maximum circumferential strain, as well as describing the spatial variability in essential parameters with higher resolution than have been previously reported. By applying this method on rats with myocardial infarction, we were able to describe distinct alterations in regional myocardial function, compared to sham-operated controls. These findings were validated against speckle-tracking echocardiography, confirming our protocol with PC-CMR as an accurate tool for investigating regional myocardial function in rats.

## Methods

### Experimental animals

Myocardial infarction was induced in male Wistar rats (10–12 weeks old, ~300 g) as described previously [18], where the left coronary artery was occluded by a silk suture. Six weeks later, cardiac imaging was performed. Inclusion criterion was visible myocardial infarction on echocardiography, and we selected both rats with small and large infarctions (infarct size range 21%-46%). Sham-operated rats went through the same procedure, except no coronary artery ligation was performed. Animals were 16–18 weeks old at the time of cardiac imaging.

The animal weights during examination were in the range 400–450 g. For evaluation of the PC-CMR method, the animals (total N = 15) were divided into two groups, one cohort to validate the method relative to echocardiography (post-MI (N = 6), sham (N = 3)), and one cohort to perform inter- and intrastudy variability analysis (post-MI (N = 4), sham (N = 2)). All animals were cared for according to the Norwegian Animal Welfare Act. The use of animals was approved by the Norwegian Animal Research Authority (ID 3284), and conformed to the *Guide for the Care and Use of Laboratory Animals* published by the US National Institutes of Health and the *European Convention for the Protection of Vertebrate Animals used for Experimental and Other Scientific Purposes* (ETS no. 123).

Anesthesia was induced in a chamber with a mixture of O_2_ and 4.5% isoflurane, and maintained during experiments by administration of a mixture of O_2_ and 1.5% isoflurane in freely breathing animals. During the CMR experiments, body temperature was maintained using heated air; and ECG, respiration rate and animal temperature were constantly monitored. Respiration was registered by an air cushion. The heart rate was kept as constant as practically possible during experiments by minor adjustments of the level of anesthesia.

### Echocardiography

Echocardiography examinations were performed on a Vevo 2100 (Visual Sonics Inc., Ontario, Canada) scanner with a 24 MHz transducer approximately one day prior to CMR scan. For the animals included in the validation study, global circumferential strain was calculated off-line by 2D speckle-tracking [19] in mid-ventricular short-axis slices. The temporal resolution of the echocardiography data was 152 frames per second.

### CMR hardware and acquisition

CMR experiments were performed on a 9.4 T/210 mm/ASR horizontal bore magnet (Agilent Technologies, Inc., USA) with a high-performance actively shielded gradient coil (inner diameter 120 mm, rise time 180 μs, max strength 600 mT/m). A quadrature volume transmit coil (inner diameter 72 mm) was used in combination with a four-channel surface receive coil array dedicated to rat heart imaging. Bipolar motion encoding gradients were incorporated into an RF-spoiled gradient echo cine sequence [9]. The acquisition employed a nine-point balanced scheme [15] encoding motion in three orthogonal directions, and was non-interleaved, that is, each encoding step was recorded separately with full temporal resolution [20]. Black-blood contrast was achieved by placing saturation slices above and below the imaging slice, applied at the end of the cine train [5]. To allow for some decay of eddy currents in the system, a short delay (τ = 250 μs) was introduced between the second encoding gradient lobe and the readout gradient.

In each animal, a mid-ventricular LV short-axis slice was planned as described by Schneider et al. [21]. Acquisition was prospectively triggered by ECG R-peak and gated for respiration by pausing acquisition during respiratory motion. 70-80 time frames were recorded covering >130% of the r-r-interval, with a temporal resolution of 3.2 ms. Overshooting the r-r-interval permitted complete coverage of the diastole in combination with black-blood saturation; and provided time for decay of gradient-induced disturbances in the ECG signal.

To reduce the impact of directional-dependent artifacts, each slice was acquired twice (corresponding to 2x signal averaging), where the second acquisition was rotated (in-plane) at least 30 degrees with respect to the first.

Key imaging parameters were as follows: TE/TR = 2.2/3.2 ms; FOV = 50x50 mm; matrix 128x128, slice thickness 1.5 mm, flip angle 7°, receiver bandwidth = 156.25 kHz; *venc* = 13.9 cm/s. Acquisition time for a complete slice was 10-15 minutes, depending on heart rate.

### CMR post-processing

The phase contrast data was extracted from the multi-receiver array coil data as described by Bernstein et al. [22], including complex-conjugated multiplication of the encoded scans with the reference scans individually for each coil element. Spatially-specific ECC was performed as previously described [13], excluding areas in the FOV subject to fold-over artifacts from analysis [15].

The data sets were subsequently semi-automatically segmented and analyzed using a purpose-written Matlab software (The MathWorks, Natick, USA). The only human inputs required during post-processing were 1) tracking of subendo- and subepicardial border at key time frames (segmentation of intermediate time frames were automatically interpolated), 2) definition of position of papillary muscles in the images, 3) definition of regions in the image subject to fold-over artifacts and 4) definition of the last diastolic time-point. Bulk cardiac motion was corrected for by subtraction of the average in-plane motion. Data points outside the myocardial mask were discarded, and the accepted data points were automatically divided into 32 segments [15]. Both the myocardial mask and segments followed the motion of the LV throughout the cycle. Finally, the velocity vector in each pixel was decomposed into a cardiopolar coordinate system, constituting of in-plane radial and tangential components and a through-plane longitudinal component [5]. The time points corresponding to peak and end-systole was automatically determined from peak global radial motion and minimum LV lumen area, respectively.

The data from the two individual acquisitions were independently processed, including division into segments using the papillary muscles as reference points, and segment-wise averaged as the last step of post-processing. To reduce low-pass filtering of the velocities following signal averaging (due to potential slight variation in heart rate), the data was normalized prior to combination. This was done by temporal stretching the data (using cubic spline interpolation) from one acquisition to the point where maximum correlation in global radial velocity between the acquisitions was achieved.

### Myocardial trajectories and strain calculations

Pixel-by-pixel motion paths were calculated from the velocity data through forward-backward-integration-based Fourier tracking [6,23], with nearest-neighbor interpolation estimating velocities at non-grid locations. Trajectories travelling out of the user-defined myocardial mask were automatically discarded, but no other signal filtering was employed. To include more data points into the analysis (since the in-slice myocardial area is larger in end-systole), the motion tracking was performed twice. This was done by extending the forward-backward motion tracking protocol to calculate motion paths with temporal origin of integration in both end-diastole and end-systole, resulting in two separate descriptions of the displacement field of the myocardium. In both datasets, circumferential strain was calculated in each of the 32 myocardial segments from the trajectories of the two adjacent segments, before the strain waveforms from the two individual trajectory tracings were averaged segment-wise.

The circumferential (Lagrangian) strain in segment *s* at time *t* was given by [24]

(1) $$Sc_{s}\left(t\right)=\frac{\left|x_{s-1}\left(t\right)-x_{s+1}\left(t\right)\right|}{\left|x_{s-1}\left(1\right)-x_{s+1}\left(1\right)\right|}-1$$

where ***x***_*s*_(*t*) is the mean in-plane position vector for all pixels in segment *s* at time *t*, and *s-1* and *s+1* refer to the two adjacent segments. As the motion paths were closed, it follows from Eq. 1 that *Sc*_*s*_(1) = *Sc*_*s*_(*t*_*ED*_) = 0, where *t*_*ED*_ is the time point corresponding to end-diastole.

### Evaluation of global and regional cardiac function

To investigate global function, peak global radial velocities and circumferential strain (*Sc*) were calculated in each animal. Furthermore, in order to evaluate regional function, the following parameters were determined:

•*Dispersion of peak motion*; the in-slice standard deviation over the 32 segments of the regional radial velocities at peak systole.

•*Dispersion of peak strain*; the segment-wise standard deviation of the regional *Sc* at end-systole.

•*Coherence of motion waveforms*: the mean temporal correlation coefficient of regional vs. global radial velocity tracings (as described by Markl et al. [25]).

•*Dispersion of motion waveforms*: the standard deviation of the above, over the 32 segments.

•*Index of dyssynchrony*: evaluated from cross-correlation delay analysis where the temporal shift in the regional velocity waveforms that maximized the correlation relative to the global motion was calculated (as described by Delfino et al. [26]). The standard deviation of the 32 delays in each animal was used as a single index of myocardial dyssynchrony.

### Validation of strain calculations

In order to validate the PC-CMR-derived circumferential strain, the global *Sc* was compared to echocardiography-derived global *Sc*. The number of temporal sampling points for a complete cardiac cycle varied between the data sets, due to differences between the techniques and animal heart rate. The dataset with lowest number of sampling points had 23 data points covering the cardiac cycle. To allow temporal paired analysis between the methods, all datasets were re-sampled using cubic spline interpolation to 23 equally spaced time points, and synchronized to peak global *Sc*.

In addition, as an internal control, the PC-CMR-derived *Sc* was compared with *Sc* estimated directly from the segmentation polygons following the borders of the subepi- and subendocardium. Here, the mean global *Sc* was estimated from

(2) $$Sc_{g}\left(t\right)=\frac{1}{2}\left(\frac{L_{\mathrm{epi}}\left(t\right)-L_{\mathrm{epi}}\left(1\right)}{L_{\mathrm{epi}}\left(1\right)}+\frac{L_{\mathrm{endo}}\left(t\right)-L_{\mathrm{endo}}\left(1\right)}{L_{\mathrm{endo}}\left(1\right)}\right)$$

where *L*_epi_(*t*) and *L*_endo_(*t*) are the lengths, at time *t*, of the polygons delineating the subepi- and subendocardium, respectively.

### Inter- and intrastudy variability

To evaluate inter- and intrastudy variability of the protocol, six animals underwent two PC-CMR examinations on separate days, one of which included two full acquisitions of the same mid-ventricular short-axis slice. Inter- and intrastudy variability in global myocardial velocities and *Sc* were analyzed using limits-of-agreement. To avoid temporal jitter, all data sets were normalized to end-systole [27].

### Statistical analysis

Student’s t-test was used for statistical analysis when comparing the dysfunctional heart to the controls, and p-values < =0.05 were considered statistically significant. Statistical analysis was performed using Matlab. Measurements are presented as mean with standard deviation in parentheses, and correlation coefficients are Pearson’s *r*.

## Results

### Animal characteristics

Animal characteristics for the rats included in the validation study are listed in Table 1. During echocardiography, the mean heart rate for the 9 animals in the validation study was 354 (35) bpm. Mean heart rate in all 15 animals during CMR experiments was 371 (31) bmp. Heart rate was not significantly different between groups. On average, the standard deviation of the heart rate in individual animals throughout the CMR examination was 10 bpm.

**Table 1:** Body and organ weights for the animals included in the validation study

	**Sham (N = 3)**	**Post-MI (N = 6)**	**t-test p-value**
**Body weight (g)**	431 (25)	406 (37)	NS
**Heart weight (g)**	1.19 (0.19)	2.20 (0.45)	0.008
**Lung weight (g)**	1.36 (0.13)	3.83 (1.24)	0.013

NS = not significant. Values are mean (SD).

### Analysis of myocardial motion

**Figure 2:**
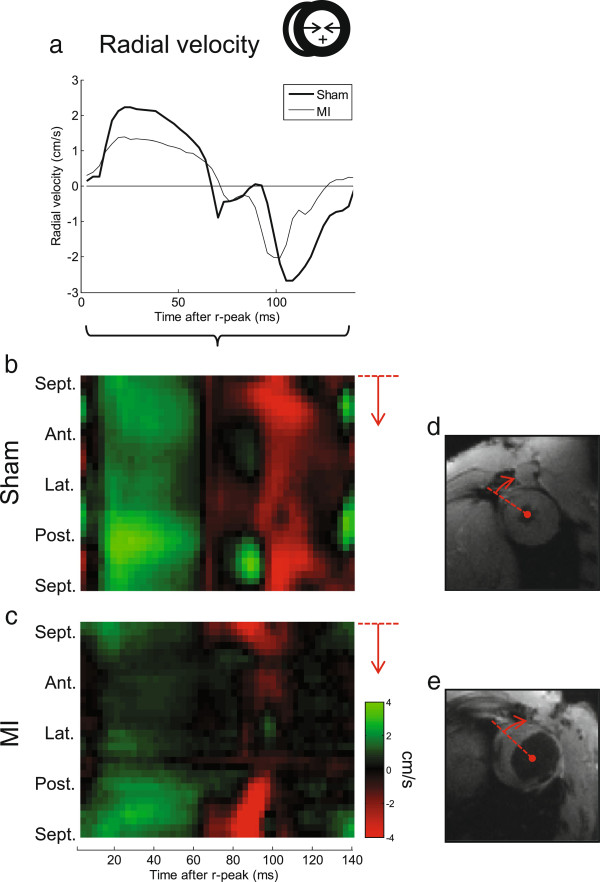
**Example of radial motion waveforms.** The global radial **(a)** in-slice velocities for two representative animals are shown, one post-MI and one sham. Note the distinct reduction in peak velocities in the diseased heart. Also, spatiotemporally resolved motion maps are displayed as colored plots **(b**-**c)** where the y-axis is circumferential position (i.e. segment; direction anteroseptal-anterior-lateral-posterior-posterioseptal), and x-axis is time after r-peak. Green color is positive radial motion (i.e. contraction), red is negative. The altered motion, especially in the anterolateral wall where the infarction is located, is clearly visible. Corresponding CMR magnitude images are shown **(d**,**e)**, illustrating the location of myocardial thinning in the infarcted heart. The line denotes the first segment and counting direction.

**Figure 3:**
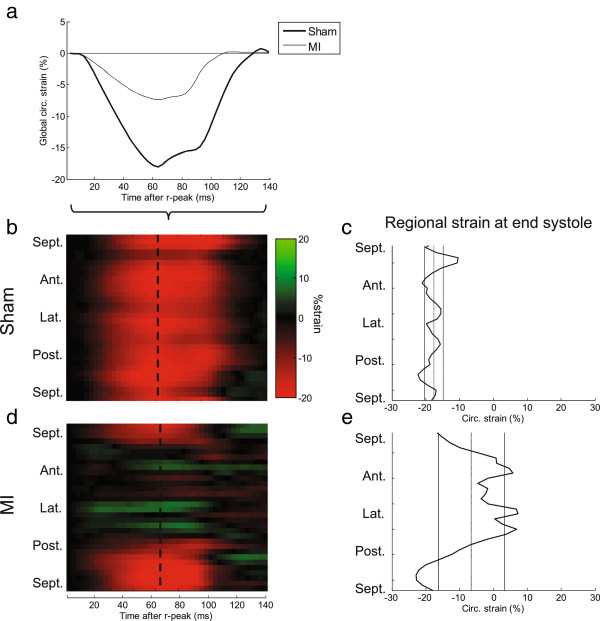
**Global and regional circumferential strain.** For the same animals shown in Figure 2, the global circumferential strain (*Sc*) is shown here as a function of time **(a)**. Furthermore, regional variation in temporally-resolved *Sc* is illustrated as color plots **(b**,**d)** where y-axis is circumferential position and x-axis is time after r-peak. Finally, the dispersion of *Sc* at end-systole (peak global *Sc*) is shown as a function of circumferential position **(c**,**e)**. Mean and standard deviation of the global *Sc* at that time point is shown as vertical lines.

Examples of radial velocities in a representative post-MI heart are shown in Figure 2, and compared to a representative control heart. Distinct alterations in both global (Figure 2a) and regional (Figure 2b-c) motion are evident in the images. The latter also demonstrate the spatiotemporal resolution of the data. Likewise, global and regional Sc are depicted in Figure 3, comparing the same post-MI and control hearts. In Figure 3c and e, the dispersions of regional *Sc* at peak global *Sc* are illustrated, that is, the profile of the line marked in Figure 3b and d. In both hearts heterogeneity in regional *Sc* is evident; however major alterations in the post-MI hearts are clearly visible.

Central parameters on myocardial function are listed in Table 2. Peak systolic radial velocity was reduced in the post-MI animals compared to the control (p < 0.001), as was global *Sc* (p < 0.001). Intragroup variation in peak diastolic radial velocity was larger in the post-MI animals compared to controls, but the mean value was not significantly different between the groups. The dispersion (i.e., the standard deviation over the segments) of regional velocities and *Sc* in peak systole and end-systole, respectively, was increased in the post-MI animals (p = 0.02 and < 0.001). The regional radial velocity waveforms in the post-MI hearts exhibited lower correlation to the respective global velocities than in the control hearts, and the standard deviation (the spread) of the correlation coefficients was likewise increased in the post-MI hearts (both p < 0.001). The post-MI hearts also exhibited increased index of dyssynchrony, identified from the spread of cross-correlation delays over the circumference (p = 0.006).

**Table 2:** Selected parameters from PC-CMR acquisition

		**Sham (N = 5)**	**MI (N = 10)**	**t-test p value**
**Peak global radial velocity (cm/s)**	Max.	2.22 (0.14)	1.41 (0.37)	<0.001
	Min.	−2.68 (0.38)	−2.64 (1.03)	NS
**Peak global *Sc *(% strain)**		−19.87 (2.28)	−6.98 (2.34)	<0.001
**Dispersion of peak motion (cm/s)**		0.58 (0.17)	0.91 (0.24)	0.02
**Dispersion of peak strain (% strain)**		5.91 (2.12)	10.40 (1.26)	<0.001
**Coherence of motion waveforms**		0.95 (0.02)	0.74 (0.09)	<0.001
**Dispersion of motion waveforms**		0.03 (0.01)	0.24 (0.10)	<0.001
**Index of dyssynchrony (ms)**		1.44 (0.37)	12.60 (7.37)	0.006

*Sc* = Circumferential strain. NS = not significant. Values are mean (SD).

### Validation of strain calculations

**Figure 4:**
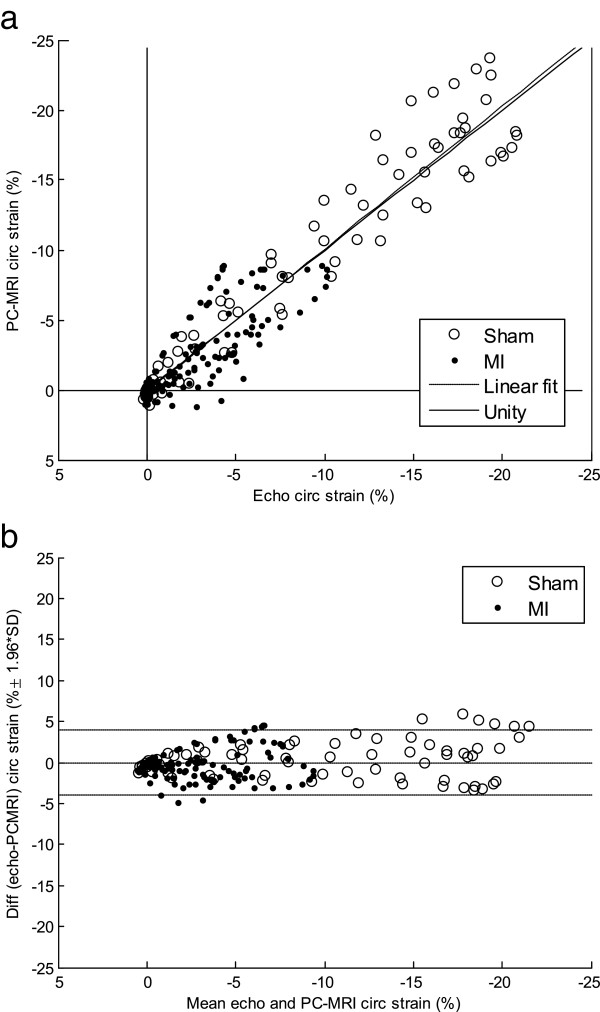
**Comparison of circumferential strain measurements from CMR and echocardiography.** Scatterplot **(a)** and Bland-Altman plot **(b)** of the data from the validation of PC-CMR-derived global *Sc* against 2D speckle-tracking echocardiography. Each animal (N = 9) had 23 equally spaced time points covering the complete cardiac cycle, producing a total of N = 207 data points. The data demonstrate small limits-of-agreement with no significant bias.

Analysis of temporally resolved global *Sc* demonstrated excellent correlation between PC-CMR and echocardiography data (r = 0.95, p < 0.001; N = 207). A linear fit revealed a close relationship between temporally resolved CMR- and echocardiography-derived data, with a slope not significantly different from 1.00 (95% confidence bounds: [0.97, 1.07], R^2^ = 0.90), see Figure 4a. Bland-Altman limits-of-agreement was −0.02 ± 3.92%strain (Figure 4b). Intra-animal analysis exhibited likewise strong correlation in all animals (mean r = 0.94 (0.08), p < 0.001 and N = 23 in all nine animals).

The PC-CMR-derived *Sc* also correlated well with *Sc* estimated directly from the segmentation polygons (r = 0.95, p < 0.001; N = 207), with limits-of-agreement −1.4 ± 4.8%. However, linear fit revealed a relationship whose slope significantly different from 1.00 (95% confidence bounds: [0.76,0.82]). Intra-animal analysis also demonstrated good correlation (mean r = 0.95 (0.05), p < 0.001 and N = 23 in all nine animals).

### Intra- and interstudy variability

**Figure 5:**
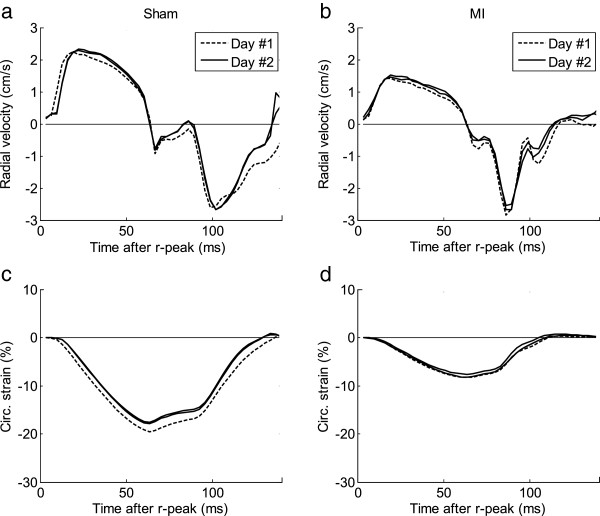
**Inter- and intrastudy variability.** Inter- and intrastudy variability in two animals (one post-MI and one sham) are shown, illustrating the correspondence between velocity waveforms and magnitude **(a**,**b)**, and *Sc***(c**,**d)**. All slices consisted of two rotations, and for intrastudy analysis the two individual acquisitions of the two slices were interleaved, attempting to reduce any physiological effects of different time spent under anesthesia.

The resulting intra- and interstudy limits-of-agreement are listed in Table 3. Intra- and interstudy variability in the actual velocity and *Sc* waveforms from a single animal are illustrated in Figure 5.

**Table 3:** Intra- and interstudy limits-of-agreement

	**Intrastudy variability**		**Interstudy variability**	
Radial velocity	0.01 ± 0.32 cm/s	0.07 ± 2.30%venc	−0.06 ± 0.75 cm/s	−0.43 ± 5.40%venc
Circ. velocity	0.10 ± 0.35 cm/s	0.72 ± 2.52%venc	0.06 ± 0.94 cm/s	0.43 ± 6.76%venc
Long. velocity	0.13 ± 0.51 cm/s	0.94 ± 3.67%venc	0.12 ± 1.15 cm/s	0.86 ± 8.27%venc
*Sc*	−0.16 ± 0.89%		−0.71 ± 1.67%	
*Sc* (single motion tracking)*	−0.15 ± 0.96%		−0.78 ± 2.31%	

Velocity variability shown in absolute values (cm/s) and percentage of the applied *venc*. Strain variability is reported in absolute values (%strain). *Sc* = Circumferential strain. N = 43x6 = 258. Values are mean ± 1.96*SD.

*: *Sc* was also calculated from motion paths using only end-diastole as temporal origin, as described in [23], without our proposed extension of also including end-systole as temporal origin.

## Discussion

In this study, we have presented a PC-CMR protocol for assessing myocardial motion in rats. Several steps were introduced in data acquisition and post-processing to optimize the protocol. PC-CMR-derived circumferential strain was validated against echocardiography, and we demonstrated that PC-CMR is capable of capturing fine details in the intricate motion of the rodent heart. The velocity and strain data exhibited distinct alterations, both globally and regionally, in the post-MI hearts vs. sham.

Our findings on global *Sc* agree well with an MR tagging study by Liu and colleagues [28], which reported mid-ventricular *Sc* in the normal rat heart as −19 (1)%. A study by the same group based on harmonic phase MR tagging [29] investigated the reduction in strain in infarcted hearts. Both studies had 15 temporal frames per cardiac cycle. In a study employing displacement-encoded CMR of infarcted mouse hearts, the circumferential strain one day post-surgery was found to be reduced from −16.4 (1.3)% in controls to −11.6 (1.8) and +4.2 (2.4)% in non-infarcted and infarcted regions, respectively [30]. They also demonstrated good correlation with MR tagging.

Distinct regional alterations in infarcted rat hearts have previously been demonstrated using speckle-tracking echocardiography [31], and our results agree well with their reported circumferential strain. That study also reported, in accordance with our findings, prominent heterogeneity in the *Sc* in healthy rat hearts. Our results are also in excellent agreement with other studies on global myocardial *Sc* in rat hearts [32,33].

A recent study by Dall’Armellina et al. [5] employed PC-CMR in studying myocardial velocities in mice, reporting corresponding findings on myocardial motion. The waveform of the global mid-ventricular radial motion is very similar between the species; however we found peak radial velocities (both in systole and diastole) in normal rats to be roughly the double of what they found in mice. Also, the regional heterogeneity of the motion around the circumference seems more pronounced in normal rats compared to mice. While this might reflect an actual variance between the species, differences could also be attributed to a fewer number of circumferential segments employed in [5].

### Validation

PC-CMR has been previously validated as a technique capable of accurately measuring velocity [4,15], displacement [16,34] and the deformation gradient of the myocardium [8]. It has been used for calculation of myocardial strain [4,7] and has been compared to MR tagging (by use of “virtual tagging”) [35]. In our study, two-dimensional speckle-tracking echocardiography (2D-STE) serves as a method for comparison. 2D-STE has been validated against tagged CMR, sonomicrometry and tissue Doppler echocardiography [36], and previously compared to DENSE CMR in mice [37,38]. Our findings demonstrate that global *Sc* in a mid-ventricular slice derived from PC-CMR correlates well with echocardiography-derived *Sc*, with narrow limits-of-agreement.

When comparing to mask-derived *Sc*, both PC-CMR and echo yielded a linear slope significantly different from 1.00, both results suggesting that the mask-derived *Sc* systematically overestimated the strain (this is also supported by the fact that the 95% confidence bounds for the linear slope of mask vs. PC-CMR and mask vs echo overlapped (data not shown), suggesting that their slopes were not significantly different). This is not surprising, as the circumferential change-of-length of the subendo- and subepicardium of the LV is expected to be a result of a combination of actual shortening of the LV both circumferentially and longitudinally, which both echo and PC-CMR would account for while the masks would not. Also, the blood volume in the myocardium itself varies throughout the cardiac cycle, contributing to the change in in-slice area of the short-axis LV images, thus not to be attributed to the actual deformation of the cardiomyocytes.

### Inter- and intrastudy variability

We found that both intra- and interstudy variability were low, the latter being comparable to previously reported values for human myocardial PC-CMR [39,40]. A source of variation in our data is suspected to be instability of animal physiology, as the data sets were acquired at different times after induction of anesthesia. Although all datasets were temporally normalized to account for varying lengths of the cardiac cycle, the actual velocities and peak *Sc* are not independent on heart rate and thus remain uncorrected.

### Limitations

Geometrically systematic artifacts as they appear in this study, along with the rotating FOV approach, only apply to Cartesian imaging.

The choice to cover more than one r-r-interval doubles the scanning time since only every second r-peak was used as trigger point. However, this allowed complete coverage of the diastolic phase in combination with black-blood preparation, and improved the reliability of the triggering by allowing decay of currents in the ECG wires. Although the acquisition time per slice in our study was quite long, it is comparable to previous reports on MR-based strain assessment [29,30]. Compared to echocardiography, the MR examination is considerably more time-consuming. Depending on study design and needs, the trade-off between data yield and acquisition time must be considered.

Although beyond the scope of this study, a direct comparison of PC-CMR-derived strain with MR tagging should be considered, the latter usually being considered as the reference standard for measurement of myocardial strain. While our study validated global strain measurements, future studies should also address comparison of evaluation of regional strain from different methods.

The algorithm for calculating circumferential strain from myocardial trajectories in this paper (Eq. 1) is rather simple compared to more complex approaches, such as spline-based deformation analysis [41]. However, the presented results suggest that our approach is appropriate, yielding accurate and reproducible results.

Since slice selection was done in the laboratory system and the heart moves longitudinally during contraction, slightly different parts of the myocardium may be imaged in different time points throughout the cardiac cycle. This motivated the choice of including end-systole as a temporal origin for estimating tissue trajectories. Compared to conventional forward-backward motion tracking, intra- and interstudy limits-of-agreements were reduced using this extension (Table 3). However, to accurately capture complex three-directional motion and thus true 3D strain, volumetric data is required [42], and should be addressed by future studies. Volumetric PC-CMR might be achieved by embedding velocity encoding gradients into conventional or accelerated 3D imaging protocols, and has been demonstrated to allow comprehensive evaluation of both blood flow and myocardial motion in humans [43-45], but not, to our knowledge, in small animals.

## Conclusion

In this study, we have presented an optimized PC-CMR protocol allowing assessment of the motion of the myocardium in rats with high detail, and provided a robust method for calculation of regional circumferential strain from the velocity data. By combining optimized slice planning, acquisition parameters and post-processing, exploration of the complex spatiotemporal pattern of *in vivo* motion and circumferential strain in the healthy and dysfunctional rat heart is feasible. We present, to our knowledge, the first study in small animals using PC-CMR to calculate strain.

## Abbreviations

2D-STE: Two-dimensional speckle tracking echocardiography; DENSE: Displacement encoded imaging with stimulated echoes; FOV: Field-of-view; LV: Left ventricle; MI: Myocardial infarction; PC-CMR: Phase contrast cardiovascular magnetic resonance; Sc: Circumferential strain; SENC: Strain-encoded CMR.

## Competing interests

The authors declare that they have no competing interests.

## Authors’ contributions

EKSE was involved in designing the study, collected, analyzed and interpreted CMR data, analyzed and interpreted echocardiography data and drafted the manuscript; JMA was involved in designing the study, provided and performed surgery on experimental animals, collected, analyzed and interpreted echocardiography data and provided critical review of the manuscript; KS and LZ were involved in designing the study, assisted in collection of CMR data, interpreted CMR data and provided critical review of the manuscript; JES was involved in designing the study, interpreted CMR data, and provided critical review of the manuscript; IS conceived and designed the study, collected, analyzed and interpreted echocardiography data, interpreted CMR data, and provided critical review of the manuscript. All authors have read and approved the final manuscript.
